# Customising the plunge-freezing workflow for challenging conditions

**DOI:** 10.1039/d2fd00060a

**Published:** 2022-08-01

**Authors:** Ian Hands-Portman, Saskia E. Bakker

**Affiliations:** a Advanced Bioimaging Research Technology Platform, University of Warwick Gibbet Hill Road Coventry CV4 7AL UK s.bakker@warwick.ac.uk

## Abstract

Grid freezing is a critical step for successful cryo-transmission electron microscopy, and optimising freezing conditions is a considerable bottleneck in many projects. To improve reproducibility in grid preparation, temperature- and humidity-controlled chambers were built into the second generation of plunge-freezers, including the ThermoFisherScientific Vitrobot and Leica GP. Since then, for most published structures, the proteins were plunge-frozen from a cold, humid environment. This provides two benefits: many proteins are more stable at 4 °C than room temperature, and both the low temperature and the humidity help control evaporation of the tiny drop of liquid. However, for optimal stability, certain samples may have different requirements. Here, we describe various (reversible) adaptations made to a Leica GP2 system to accommodate several samples with special handling requirements: a protein that is sensitive to both light and oxygen, a sample that needs to be kept at 37 °C throughout the plunge-freezing process, and a method to freeze a polymer that gels at 37 °C in its gelled state. While some of these methods are specific to these specimens, we hope sharing the ideas behind them will help people who are dealing with tricky protein samples.

## Abstract 2

Modifications to a commercial plunge-freezer are described, enabling temperature-sensitive, light-sensitive and oxygen-sensitive samples to be frozen for analysis by cryo-TEM.

## Graphical abstract

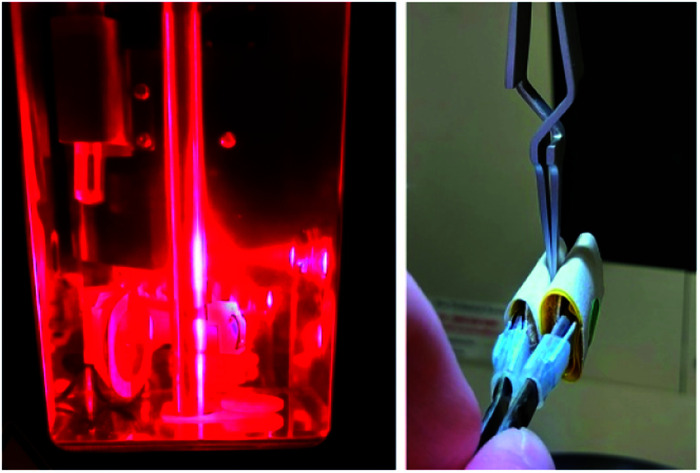

## Introduction

The preparation of biological specimens for transmission electron microscopy (TEM) must stabilise the samples so they can withstand the vacuum within the microscope. Biological samples, by their nature, contain a large amount of water, which serves not only as a solvent but also as a physical support. As such, it plays an important role in the structural integrity of macromolecules and complexes, as well as other biological structures such as membranes. Dramatic changes in structure and integrity may occur when biological samples are dried for room temperature observation.^1^

The development of plunge freezing enabled TEM imaging of biological samples in the presence of water. The main innovation was vitrification, *i.e.* cooling a sample so rapidly the water molecules become immobilised in their solution state.^2^ This prevents the formation of ice crystals, which cause severe damage to most biological structures.^1^ Once frozen, the samples are maintained at cryogenic temperatures during storage, transfer and imaging. This continuous cooling of the samples during imaging also reduces damage in the sample,^3^ although low-dose imaging^4^ is still required to reduce melting of the sample during imaging.

The first-generation, mostly manual, plunge-freezers provided a way to hold the grid during sample application and blotting, as well as a mechanism for accelerating the grid into the cryogen container. Blotting was achieved by pressing filter paper manually onto the grid, or assisted by pressurised gas from one or both sides of the grid; plunging was by gravity, assisted by the weight of the mechanism, or again assisted by pressurised gas.^5,6^

A second generation of computerised plunge freezers was developed commercially, aiming to provide increased reproducibility between samples and individuals. The blotting time and pressure, as well as the application of the filter paper to the grid, are more precisely controlled in these plunge-freezers. Environmental control chambers also provided improved reproducibility despite variations in lab temperature or humidity due to weather conditions and geographical location.

Often, purified proteins are kept in fridges and ice boxes both during and after purification to slow down deterioration of the sample. The commercial plunge-freezers enabled users to maintain the protein at 4 °C and high humidity during freezing. These conditions also contribute to the plunge-freezing process itself by helping prevent evaporation of the thin film of water during the time it takes to go from blotting to vitrification.

With the exception of certain extremophile organisms, however, the low temperature is not “native-like”, and some proteins do not respond well to colder conditions. For example, microtubules assemble only at higher temperatures and have long been known to depolymerise when kept at 4 °C.^7,8^ Recently, temperature-dependent effects in a thermophilic enzyme were resolved by cryo-EM after plunge-freezing from different starting temperatures.^9^ Other proteins may also benefit from higher temperatures.

The effect of higher temperatures on structures is of interest to those developing materials intended for *in vivo* use, such as drug delivery mechanisms or wound dressings. One of the examples discussed below is a thermoresponsive hydrogel, which is liquid at room temperature but gelates quickly when exposed to temperatures of 37 °C and higher. These can be administered as liquids, but form a stable gel once injected. In drug delivery, this can help with the local controlled release of compounds, reducing systemic side effects, while in tissue engineering the polymer acts as a scaffold during cell proliferation.^10^ The commercial plunge-freezers offer a range of conditions, *e.g.* the Leica GP2 allows chamber temperatures from 4 to 60 °C and humidity up to 95%, so they enable freezing directly from temperatures other than 4 °C.

More recent developments in grid preparation aim to improve reproducibility by further automating the process, which reduces variation between users. They also reduce the amount of sample required by eliminating the blotting step by printing or spraying the sample onto the grid^11–13^ and using self-wicking grids.^13^

In addition to maintaining the structural integrity, cryo-TEM makes it possible to see the internal structure of a sample. This enables the full 3D reconstructions of proteins, but also allows for the visual confirmation of internal features, such as the encapsulation of materials inside lipid vesicles. The methods developed for biological specimens can also be applied to soft materials, which exhibit similar challenges in terms of beam damage and structural integrity loss when imaged using the dry state TEM methods commonly used for specimens in material sciences. Therefore, grid freezing is an essential step in preparing samples for use in cryo-electron microscopy.

## Experimental setup modifications and results

### Using a plunge-freezer

A Leica GP2 plunge-freezer was used for all these experiments. In standard conditions, the GP2 is operated as follows:

(1) The user picks up the grid in tweezers and mounts the tweezers in the equipment.

(2) The tweezers are enclosed by the environmental chamber, with the ethane positioned underneath.

(3) The user applies a 3–8 μL sample to the grid through one of the sample ports in the chamber, using a micropipette.

(4) A blotting sequence and optional post-blot waiting step are carried out.

(5) The grid is plunged into the liquid ethane.

(6) The user transfers the grid into a storage container.

Settings, such as the blotting time, are controlled *via* the touch screen that sits on the bench next to the machine. The different steps in the process can be initiated using the touch screen, or using a foot pedal to initiate the next action in the sequence.

### Overcoming the quick gelation of temperature-dependent hydrogel polymers

Hydrogels are three-dimensional physical networks of polymers in aqueous media. In thermoreversible hydrogels, the networks are formed in response to temperature variation, through physical interactions between the polymer chains.^10,14^ The samples of interest are liquid at room temperature, consisting of small particles, but gel rapidly at 37 °C through the formation of aggregates large enough to cause a milky or opaque appearance. Due to the physical, rather than chemical, nature of these bonds, the gelation is reversible upon cooling to room temperature.

**Fig. 1:**
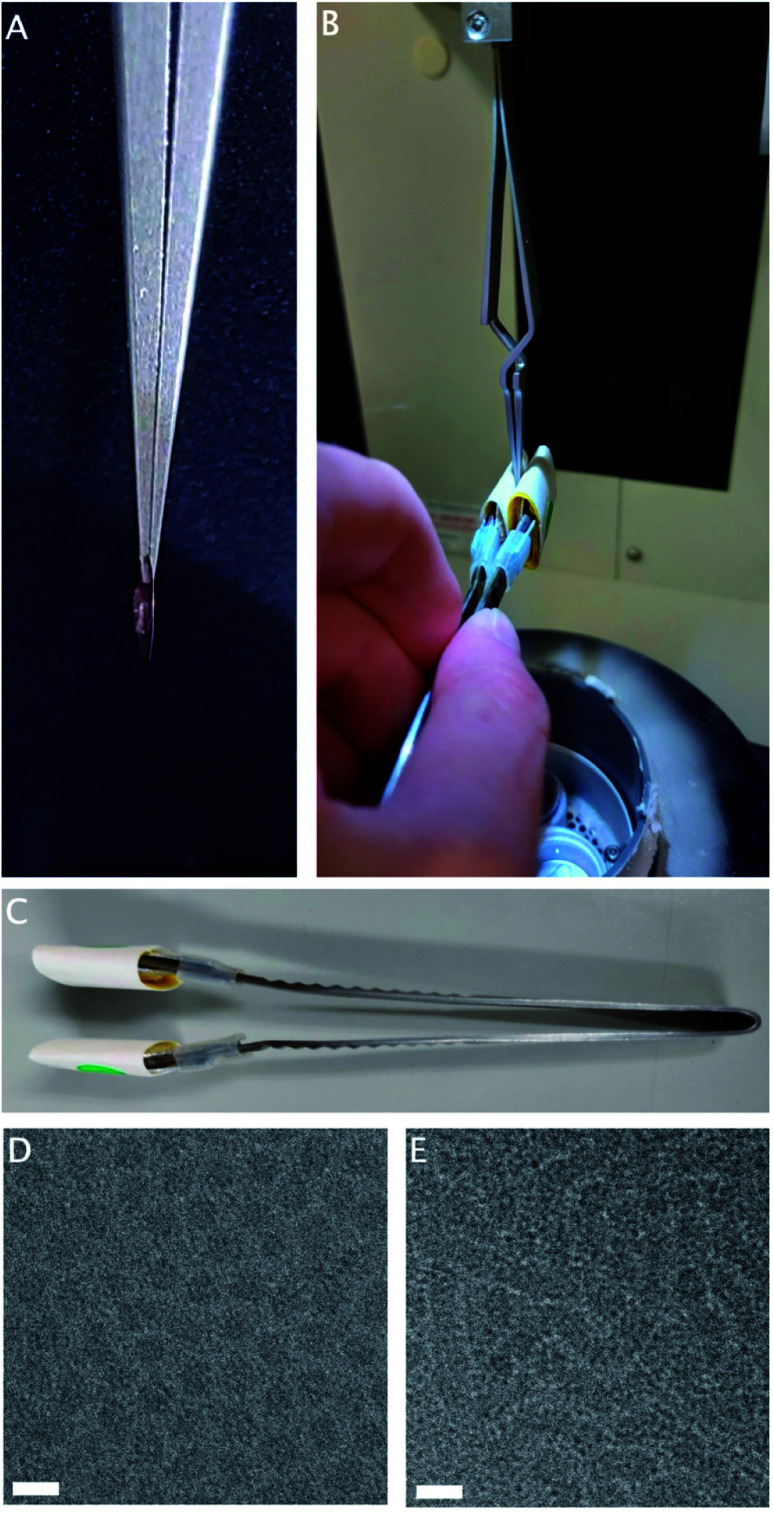
(A) Insufficient blotting of a gelled sample. A thick gel can be seen on the grid after blotting. (B) Manual blotting of the sample before lowering the environmental chamber using custom-made blotting forceps. (C) Custom-made blotting forceps with widened areas near the tip to affix filter paper. (D) Electron micrograph of gel sample frozen from room temperature, showing individual round micelle-like structures. (E) Electron micrograph of gel sample frozen from 37 °C, showing tubular structures. White bar 20 nm.

While the chamber temperature of the plunge-freezer is adjustable, rapid temperature changes within the chamber are not possible. If the normal procedure is followed with the chamber at 37 °C, this sample reaches full gelation before blotting has completed (Fig. 1A), resulting in incomplete blotting and ice that is too thick for imaging. However, as the sample is liquid at room temperature, it is possible to perform a manual blotting step of the sample before lowering the heated chamber, and allowing post-blotting incubation in the chamber at 37 °C to complete gelation (Fig. 1B).

Double-sided manual blotting was performed using modified forceps, fitted with cushioned metal tabs to expand the surface area and reduce the local pressure on the grid, covered with filter paper (Fig. 1C). To perform blotting, the tabs are pressed to both sides of the grid. Once the chamber is lowered, a generous incubation time allows full gelation to take place on the thin layer of remaining sample, before plunging is completed as usual.

The small volumes involved in plunge-freezing provide very little resistance to temperature change, therefore strict temperature regulation is hard to maintain while transferring samples from tubes to grids. A separate sample consists of small particles at RT, but at 37 °C forms larger aggregates. These aggregates are sufficiently large to cause a milky or opaque appearance.

Without precautions, the mass of the room-temperature pipette tip will cause visible clearing of the solution as the temperature drops below 37 °C, even in the small amount of time it takes to transfer the heated solution to the heated environmental chamber of the plunge-freezer.

Pre-heating the pipette tips in dry glass beads, as well as keeping the solution incubated at 37 °C, helps prevent the visible clearing of the solution as a result of a temperature drop.

### Freezing light-sensitive proteins

Cobalamin-containing enzymes are found in bacteria involved in the breakdown of organic matter. Adenosylcobalamin (coenzyme B12) serves as the cofactor for this group of enzymes that catalyze unusual rearrangement or elimination reactions. Crystal structures indicate that a photolytic cleavage of the cofactor can happen, both in crystals and in solution. Therefore, the preparation of cryo-EM grids will have to be carried out under red light conditions.

The cofactor acts as the initiator of reactive free radicals, which are needed for the enzymatic reaction. However, it is not clear how the enzymes activate the cofactor and control the radicals once these are generated. In addition, catalysis requires a large domain movement from an “open” to a “closed” conformation. This domain movement has been modelled but no high-resolution structures have been produced as the movement is prevented by the crystal lattice.^15^

To provide insight in the reactions performed by this class of enzymes, high-resolution structures are needed of the different reaction states. Cryo-EM can accommodate the large domain movements in a way that crystallography cannot, as well as being able to sample multiple structural conformations from the same grid.^16^

**Fig. 2:**
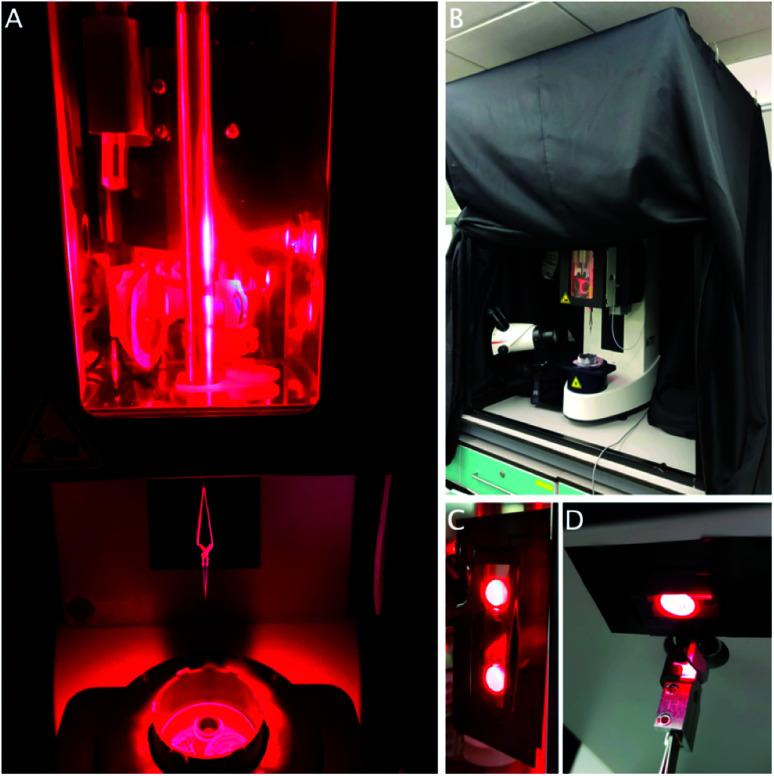
(A) Leica GP2 environmental chamber and under-chamber lights covered in a red lighting filter. (B) Blackout curtain over the metal cage. (C) Close-up view of the chamber light with a red filter. (D) Close up view of the under-chamber light.

The Leica GP2 has two built-in white LED light sources to facilitate the freezing process. One is positioned inside the chamber to help with application of the sample; the second one is placed under the chamber to facilitate grid transfer (Fig. 2A). While these lights can be controlled, in our case, not all the lab overhead lighting can be turned off as it functions as emergency lighting, so instead a cage was built and fitted with a blackout curtain to exclude environmental light (Fig. 2B).

After excluding all room light, the built-in white LEDs were covered with red filter gels to block any other wavelengths, while other sources of light were covered in black electrical tape (Fig. 2C and D). The touch display controlling the settings was put outside the curtain and the foot pedal was used to initiate the next step in the process. During the freezing process, the operator will be surrounded by the curtain. It is imperative to use a personal oxygen alarm as the curtain may hinder air flow around the equipment, resulting in reduced oxygen concentrations, although this was never observed during our experiments. The sample was otherwise applied and frozen using the procedure described above.

Opening or removing the curtain and removing the red gels easily returns the equipment to a state suitable for other users, which is important in a multi-user environment. The cage is sufficiently unobtrusive and it can remain in place until completion of the project, when it can be taken apart for storage. This method can easily be modified for other wavelengths using different coloured gels. A separate room where the light can be turned off would obviously be an improvement, although it may still be necessary to block or exclude light from emergency exits and other equipment (Fig. 2).

### Freezing oxygen-sensitive proteins

Despite the ubiquity of oxygen in the atmosphere, many organisms, proteins and processes are sensitive to the presence of oxygen. Obligate anaerobic organisms, such as those from deep-sea environments or used in biogas plants, cannot grow at all in the presence of even small amounts of oxygen.^17^ Medically relevant facultative anaerobes can cause infections under anaerobic conditions in the body when they colonise places without an oxygen-rich blood supply.^18^ Studying these organisms and their processes requires oxygen-free conditions to provide a native-like structure.

The cobalamin-containing enzyme described above is found in the anaerobic bacterium *Clostridium sticklandii* and is sensitive to the presence of oxygen, in addition to its sensitivity to light. Adding the substrate to a purified protein solution in an aerobic environment leads to the rapid and irreversible formation of hydroxycobalamin, causing depletion of the cofactor^19^ and increased structural flexibility.^15^ Therefore, the ligand-bound structure can only be resolved under anaerobic conditions.

Using the environmental chamber built into the plunge-freezer, a small positive pressure of oxygen-free nitrogen gas can be maintained. The ports required for sample application and grid plunging prevent a dangerous build-up of pressure, although spare ports should be closed off to prevent the mixing in of atmospheric air. The greatest challenge in this setup is maintaining the anaerobic conditions during sample transfer.

The usual method of applying the sample to the grid prior to plunge-freezing uses micropipettes. These typically work by air displacement and have no barrier between atmospheric conditions and the sample. The shape of the tip also causes mixing within the sample, which means any oxygen present will be mixed evenly throughout.

Positive displacement pipettes, often used for pipetting viscous or volatile samples, could be used instead, but still require fully opening the sample container due to the physical size of the tip.

For air-sensitive transfers, it seems useful to look to methods that routinely analyse gaseous samples, such as gas chromatography. Manual injection in a gas chromatography system is done using a precision syringe, which is also gas tight. This way, samples can be prepared in an anaerobic chamber. For storage and transport, glass vials with a screw-on septum cap provide a gas tight option that, once filled with the nitrogen atmosphere of the anaerobic chamber, should keep the sample from deteriorating. The septum can be pierced with the gas chromatography syringe to aspirate the sample without introducing oxygen into the vial. The graduated syringe is used to apply the sample directly to the grid, within the nitrogen atmosphere of the environmental chamber of the plunge-freezer. Blotting and plunging can then proceed as usual.

## Discussion

The commonly used starting point for plunge-freezing, 4 °C and high humidity, has been extremely successful in providing many high-resolution protein structures. However, this work shows how some samples cannot be successfully imaged using these standard conditions. The four different conditions described here resulted from samples provided by three different users of our multi-user facility, illustrating how different conditions may be required at approximately the same time, while keeping the equipment available for use at standard conditions by other users. This emphasises the need for any modifications to the process to be easily implemented during the optimisation stage. It also requires the user to have obtained sufficient information about their samples to help determine the optimal conditions.

Once frozen, cryo-TEM grids must be kept under −160 °C to prevent phase transitions from vitreous to crystalline ice.^1^ For all the samples discussed in this work, it is assumed that the storage conditions required to prevent this transition will be sufficient to prevent other changes in the sample as well, despite small temperature changes and necessary brief exposures of the grid to light and atmosphere during sample transfers.

### Validating the conditions

To ensure that valuable microscope time is used effectively, it is necessary to carefully validate the sample conditions during the freezing process. An incomplete structural change will limit the resolution by providing fewer particles, while an unsuccessful one will result in a structure that is identical to that of the standard conditions. A visual indicator of the correct conditions is absolutely invaluable for the validation process. Due to the small sample volumes involved in plunge-freezing, it is extremely challenging to measure the physical properties, such as the temperature or pH, of the sample directly. A visual change to the sample is more easily observed. The indicator must react quickly to the change in properties, and ideally be observable without interference.

The temperature-sensitive nanoparticle aggregation was easily observed even in very small volumes. It was obvious during the pipetting step when the temperature had dropped too far as the sample immediately went clear. By contrast, the gelation of the temperature-sensitive hydrogels was harder to observe as the gel and the liquid are optically similar. Gelation was only observed through tactile interactions such as the blotting process, attempted pipetting, or agitating with a pipette tip. For detecting the presence of residual oxygen, colorimetric redox indicators can be used. One safe and effective redox indicator is methylene blue, which is commonly used for redox demonstrations in school chemistry labs.^20^ The reduced dye is colourless and turns blue in the presence of oxygen. Commonly the solution includes glucose as a reducing agent, which makes the reaction reversible. In cryo-TEM, glucose causes an increase in the beam-sensitivity and radiation damage of the sample, so other reducing agents would be more appropriate.

For a more sensitive measurement, indigo carmine has long been used to detect dissolved oxygen in water and beer.^21^ Depending on the levels of dissolved oxygen, its colour varies between yellow for no oxygen, through orange and red to blue.^22^ Other available dyes include phenosafranine^20^ and resazurin.^17^

Only small amounts of any of these dyes are required for the detection of oxygen, and ideally they would be included in the sample buffer. This way, they serve as a validation method for the entire process and problems with sample transfer steps can quickly be diagnosed, saving time on further steps in the experiment. However, some of the dyes are pH sensitive, which may make them incompatible with some sample buffers. Reversibility of the reaction may not be desired if it is to serve as a readout for changes, although if compatible, a small amount of reducing agent in the sample buffer may prevent large changes and help make the experiment viable.

### Wider applications

In one of the experiments described here, we excluded light at specific wavelengths to prevent deterioration of the enzymatic co-factor. However, the method can also be used to prevent the premature initiation of light-sensitive reactions that are activated by visible light. Enzymatic reactions can be initiated and synchronised by adding covalently bound photolabile groups to the sample, either the protein or the ligand, which will initiate the reaction upon irradiation with an appropriate wavelength. This process, generally called photocaging, has been applied in crystallographic experiments to obtain the structures of reaction intermediates, as well as cell biology.^23^ In crystals, the crystal lattice may prevent large-scale domain movements, as well as limiting diffusion of the ligand. This can lead to incomplete conformational changes and incomplete occupancy of the ligand, resulting in reduced confidence in the resulting structure. In plunge-freezing, the photoactivation takes place while the protein is in solution, immediately before freezing, which allows enough time for rapid domain movements.

A method has previously been described for UV-activated samples, where the sample is photo-activated by illuminating the area above the ethane container, resulting in a rapid freezing after activation.^24^ A photo-labile source of protons was exposed to UV light to rapidly lower the pH and capture a low-pH intermediate state of an acid-sensing ion channel. However, this method would need to be modified to exclude external light for samples activated by visible wavelengths to prevent premature activation. An LED source could be added to the dark-modified GP2 for a similar result in such cases. The photo-activation of samples during high-pressure freezing has been possible for some time.^25^

The modifications to the workflow and equipment described here can easily be adapted to other plunge-freezing devices, including manual ones. More automated vitrification systems may not be able to vitrify certain samples successfully, depending on the technology they use. Viscous samples or those with a surface tension different from that of water, especially, may prove troublesome when it comes to using capillary application or self-wicking grids, which rely on the sample having the physical properties of an aqueous solution. Therefore, those samples will likely continue to require the adaptability and flexibility of the more manual or semi-automated plunge-freezing devices.

## Conclusions

In this work, we describe several reversible modifications to the workflow of the commercially available Leica GP2 plunge-freezer. These modifications enabled temperature-sensitive, light-sensitive and oxygen-sensitive samples to be plunge-frozen for analysis by cryo-TEM. Although these methods were developed specifically to overcome the challenges posed by the particular conditions required for these samples, they may be more widely applicable to other samples. Additionally, these methods can be adapted or combined depending on the needs of the sample. This will help explore samples in a wider range of physical conditions by cryo-TEM. This work shows how the flexibility of a manual plunge-freezer can be an advantage when dealing with challenging samples. Whether this flexibility extends to more automated systems remains to be seen.

## Author contributions

SEB: conceptualisation, investigation, methodology, writing. IHP: methodology, writing.

## Conflicts of interest

There are no conflicts to declare.
